# Osteopathic Manual Therapy for Infant Colic: A Randomised Clinical Trial

**DOI:** 10.3390/healthcare11182600

**Published:** 2023-09-21

**Authors:** María del Mar Martínez-Lentisco, Manuel Martín-González, Juan Manuel García-Torrecillas, Eduardo Antequera-Soler, Raquel Chillón-Martínez

**Affiliations:** 1 Andalusian Health Service, Almería Health District, 04002 Almería, Spain; 2 Department of Nursing Science, Physiotherapy and Medicine, University of Almería, 04120 Almería, Spain; mmg189@ual.es (M.M.-G.); easantequera@gmail.com (E.A.-S.); 3 Torrecárdenas University Hospital, 04009 Almería, Spain; 4 Emergency and Research Unit, Torrecárdenas University Hospital, 04009 Almería, Spain; juanma.gator@gmail.com; 5 CIBER de Epidemiología y Salud Pública (CIBERESP), 28029 Madrid, Spain; 6 Instituto de Investigación Biosanitaria Ibs, 18012 Granada, Spain; 7 San Isidoro University Center, University of Pablo Olavide, 41092 Sevilla, Spain; rchillon@centrosanisidoro.es

**Keywords:** infant colic, manual therapy, osteopathy, physiotherapy, pediatrics

## Abstract

Background: Infant colic is a multifactorial syndrome for which various therapeutic strategies have been proposed. In this study, we evaluate the effectiveness of osteopathic manual therapy in treating symptoms related to infant colic. Method: A prospective, randomised, blinded clinical trial was conducted of patients diagnosed with infant colic. The treatment group were given osteopathic manual therapy, and their parents received two sessions of counselling. The control group received no such therapy, but their parents attended the same counselling sessions. The non-parametric Mann–Whitney U test was applied to determine whether there were significant differences between the groups for the numerical variables considered. For the qualitative variables, Fisher’s exact test was used. The threshold assumed for statistical significance was 0.05. Results: A total of 42 babies were assigned to each group. Those in the experimental group presented less severe infant colic with a trend towards statistical significance after the first session (*p* = 0.09). In sucking, excretion, eructation and gas there were no significant differences between the groups. Crying was a statistically significant dimension both after the first intervention (*p* = 0.03) and two weeks after (*p* = 0.04). Regurgitation values were significantly lower in the experimental group during the three weeks of follow-up (*p* = 0.05). Values for sleep were lower in the experimental group, but the differences were not statistically significant. In both groups, colic severity decreased over time, with no side effects. Conclusions: Treatment with osteopathic manual therapy alleviates the symptoms of infant colic and could be recommended for this purpose from the onset of the condition.

## 1. Introduction

Infant colic is a syndrome reflected by paroxysmal, persistent and irritable crying in a healthy baby, together with meteorism and irritability [1,2,3,4,5]. It is often accompanied by regurgitation [5,6,7] and difficulty in achieving rest and sleep [8,9].

The condition has been diagnosed in up to 40% of healthy infants during the first three months of life [1,10,11]. It is a common reason for visits to the paediatrician [12], accounting for 10–20% of consultations for infants aged between 2 weeks and 3 months [13], and to hospital emergency departments during the first months of life [12,14,15]. However, the interpretation of crying is subject to parental perception [16] and its real presence may be greater. In case of doubt, parents are encouraged to seek professional help, as prolonged severe crying may reflect a health problem [17].

The aetiology of infant colic is complex and multifactorial [18]. Organic hypotheses include cow protein allergy [19], altered oesophageal and gastrointestinal motility [20] or gastro-oesophageal reflux [21].

The main behavioural hypotheses proposed for infant colic are related to parental stress and postpartum depression [22,23], difficulties in establishing a satisfactory feeding pattern, the immaturity of rest and sleep patterns and/or problems related to the response to physiological needs [22,24].

Treatment strategies are usually focused on organic hypotheses, especially the presence of gastrointestinal gas [25,26]. In addition, changes in infant feeding have been recommended, with the incorporation of hypoallergenic formulas [27,28] or probiotics [29,30].

Controversy may arise in parental decision-making concerning feeding behaviour, with respect to breastfeeding, formula feeding or mixed feeding. As a potentially valuable alternative, manual therapy is becoming increasingly accepted as a treatment for infant colic, due to its non-pharmacological nature [20,31,32]. Recent studies suggest that manual therapy can help reduce crying time and improve sleep patterns [21,33]. Within this approach, osteopathic manual therapy is recommended for the treatment of gastrointestinal disorders [34,35,36] by addressing imbalances in the autonomic nervous system caused by tension and altered tissue tone, both at the base of the skull and in the fascial system [37,38,39], and by functional alterations of the spinal column [9]. In a global way, it can be assumed that the purpose of osteopathy is to diagnose and treat the mobility dysfunctions of the tissues of the human body, which cause disorders and disturb the state of health of the organism, so in osteopathy the musculoskeletal, visceral and cranial systems are inseparable and affect each other [40]. Osteopathy emphasizes the structural and functional integrity of the body and the body’s intrinsic tendency to self-heal [41]. 

Studies have reported great heterogeneity in how manual therapy is provided [42]. To date, there is no consensus as to which models or techniques are most effective [21,43,44]. The intervention protocols applied are often incompletely described [35,45] and the results obtained are mostly final outcomes reported by parents, i.e., intermediate results are not provided [43].

In view of the above considerations, the aim of this study is to evaluate the efficacy of osteopathic manual therapy for infant colic, using a specific manual therapy protocol, paying attention to the clinical variables of crying, sleep and regurgitation for their clinical implication in infant colic, describing the possible differences between the evolution of infant colic in both groups and examining whether there is a relationship between the study variables, such as type of feeding and type of delivery, and the severity of the condition.

## 2. Materials and Methods

A randomised, single-blind clinical trial was conducted on a sample of babies diagnosed by their paediatrician with infant colic. These patients and their parents were referred to the physiotherapy room at the collaborating health centres (Níjar and Nueva Andalucía, both in the province of Almeria, Spain) where they were invited to participate in the study. This study was registered in the International Database of Clinical Trials (clinicaltrials.gov) (NCT 03326297).

### 2.1. Participants

Inclusion criteria: infants less than 2 months old, with more than 37 weeks’ gestation and presenting with episodes of inconsolable crying. Exclusion criteria: presence of any other clinical sign or organic pathology justifying the infant’s crying, babies diagnosed with other pathologies that justify infants’ crying, such as reflux, Sandifer syndrome, food intolerances or neurological syndromes and premature babies of less than 37 weeks of gestation, who may have other concomitant pathologies or greater immaturity that justifies crying.

### 2.2. Sample Size

The necessary sample size was determined as follows, using G*Power 3 software (RRID:SCR_013726). For a statistical power of 90%, to test the null hypothesis H₀ (that the two populations compared are equal) by applying the Wilcoxon–Mann–Whitney non-parametric test for two independent samples, assuming a significance level of 5%, for an experimental group with a mean size (standard deviation) of 48.07 (15.35) units and a control group of 57.87 (8.91) units, we calculated that 38 infants should be included in the experimental group and 38 in the control group. This was based on the study by Keefe et al., 2006 where they used osteopathic manual therapy for the treatment of infant colic [46]. To compensate for an expected dropout rate of 10%, a total of 42 infants per group were recruited.

### 2.3. Randomisation

Using Epidat 3.1 [47], the participants were blindly assigned by simple randomisation to either the experimental or the control group. In both groups, the physiotherapist maintained direct and continuous manual contact with the infant during the intervention, so parents were unaware of whether or not manual therapy was being applied. Similarly, the paediatricians were blinded because they were not present during the intervention. Neither the family nor the paediatricians involved were aware of which groups the infants belonged to. 

### 2.4. Intervention 

The interventions were performed in the physiotherapy room and in the paediatrician’s office when it was free for use. In every case, the treatment was provided with full respect for the baby’s tolerance and comfort. All appropriate hygienic measures were taken, and the room temperature was sufficient for the infants to be dressed only in underwear or nappies. The procedure lasted approximately 60 min.

The physiotherapist who performed the interventions had official training in osteopathic manual therapy, recognised by the Scientific European Federation of Osteopaths, and over ten years’ experience in this field. Thus, professional competence was assured and variability minimised.

Both the experimental and control groups received counselling sessions on two occasions, with the second 15 days after the first. Both groups were given health education recommendations and ergonomic and postural advice, such as how to relax the baby in the event of colic crisis, appropriate techniques for feeding and breastfeeding to avoid air being swallowed, and how to respond to the baby’s demands.

#### Osteopathic Manual Therapy

As well as the above counselling sessions, the infants in the experimental group received a protocol of osteopathic manual therapy, focused on the cranial sphere with the following specific actions: de-imbrication of all cranial sutures, spheno-basilar decompression, re-balancing (using the Sutherland technique) of the SEB dysfunctions, the parieto-squamous suture and the intra-osseous torsion of the occiput and temporal scales, de-enclavation or lifting of the frontalis (using the parietal lift technique), application of the Lambda functional technique and of the “opening” technique to the occipitomastoid suture. In addition, appropriate techniques were applied to the thoracolumbar hinge and diaphragm, to achieve functional anterior–posterior balancing and to stretch the muscle fibres of the diaphragm. Finally, visceral techniques were applied, targeting the course of the colon, with special attention to the areas of greatest tissue tension, in the sphincter of Oddi, the ileocecal junction, the cardia, pylorus and sigmoid colon. In the control group, the therapist also applied manual contact to various anatomical areas, but without performing the aforementioned techniques, in order to ensure the blinding of the parents to the procedure.

### 2.5. Data Collection

In the first consultation, the parents were asked to provide signed, informed consent to participate and then to complete a data collection sheet and a questionnaire on infant colic severity (ICSQ) [48] based on seven dimensions: sucking, excretion, eructation, gas, crying, regurgitation and sleep. Questionnaire data were collected prior to treatment (baseline, T0), at one week after the first treatment (T1), at two weeks (when treatment was performed again, T2), at three weeks (T3) and at three months of age (T4).

### 2.6. Data Analysis

In our analysis, each quantitative variable is expressed as the mean and the standard deviation, or as the median and the interquartile range. The qualitative ones are expressed via frequency tables and percentages, with 95% confidence intervals. In most cases, these values are calculated by the normal method, and otherwise by the exact binomial method, but only where expressly mentioned. After confirming the normality of the distribution, the non-parametric Mann–Whitney U test was applied to assess differences between the control and intervention groups, with respect to the numerical variables. For the qualitative ones, Fisher’s exact and the chi-square test were used. In all cases, the threshold of *p* ≤ 0.05 was assumed for statistical significance. The repeated measures mixed effects model was used to discern whether there were different long-term measurements for each of the groups in the different dimensions of the questionnaire. All calculations were performed using STATA v12 statistical software and SPSS version 26 (IBM Inc., Armonk, NY, USA).

## 3. Results

**Figure 1:**
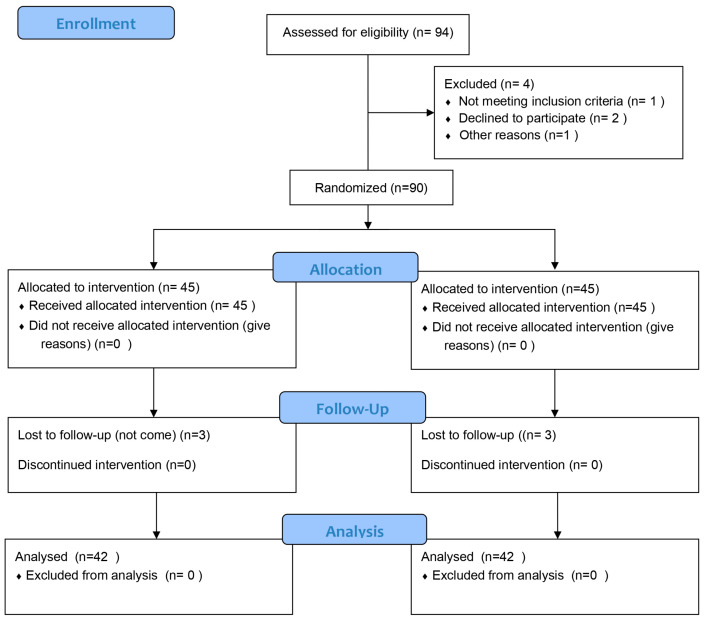
CONSORT flow diagram.

Figure 1 shows the CONSORT flow diagram.

Table 1 summarises the descriptive data obtained for the experimental and control groups. The characteristics observed were homogeneous except for the consumption of products to alleviate colic, which was greater in the control group than in the experimental group (95.24% vs. 80.95%, *p* = 0.045).

There was no relationship between the severity of infant colic and the type of infant feeding (*p* value 0.74) or the type of delivery (*p* value 0.31). 

As shown in Table 2, the severity of colic was lower in the experimental group than in the control group throughout the follow-up period. Although the differences trended towards statistical significance (*p* = 0.09) after the first treatment, the differences were not statistically significant overall. Furthermore, colic severity decreased in both groups over time.

Concerning the differences between the evolution of infant colic severity, with the repeated measures design in both groups, the severity values were statistically significant both over time (*p* < 0.001) and in the time–group interaction (*p* < 0.05). Within-group differences (taking the mean of the five severity measurements) were not statistically significant (*p* = 0.18).

The following results were obtained for each of the questionnaire dimensions presented in Table 3. For crying, the experimental group presented lower values at T1 and T2. At both time points, the differences were statistically significant (*p* = 0.03 and *p* = 0.04, respectively). For regurgitation, the values were lower in the experimental group for T1, T2 and T3 (*p* = 0.05, *p* = 0.05 and *p* = 0.04, respectively). Although values were also lower at T0, these are not significant differences and it was observed that crying at T1 was reduced in the experimental group and remained similar in the control group. Regurgitation decreased after the second treatment in the experimental group and was similar at T4 when the colic symptoms were considered resolved. No statistically significant differences were observed for sucking, excretion, eructation or gas. For sleep, although the values were lower in the experimental group at T1, the differences were not statistically significant. A more favourable evolution was observed in the experimental group, that is, the infants obtained better scores in regurgitation and crying, mainly for T1 and T2 (after the first intervention). For the other dimensions, no significant differences were observed between the two groups.

In the repeated measures design for each of the dimensions of the questionnaire, no significant differences were observed between the groups, but significant differences were observed in the group–time comparison. As previously mentioned, the levels of the dimensions of the questionnaire decreased to normal levels more rapidly in the experimental group than in the control group. This difference is important for the first time periods considered (T1–T3). By three months of age (T4), the problem of colic is usually resolved and therefore similar results between the two groups are to be expected. Nevertheless, the experimental group presented lower levels throughout the intervention process.

**Figure 2:**
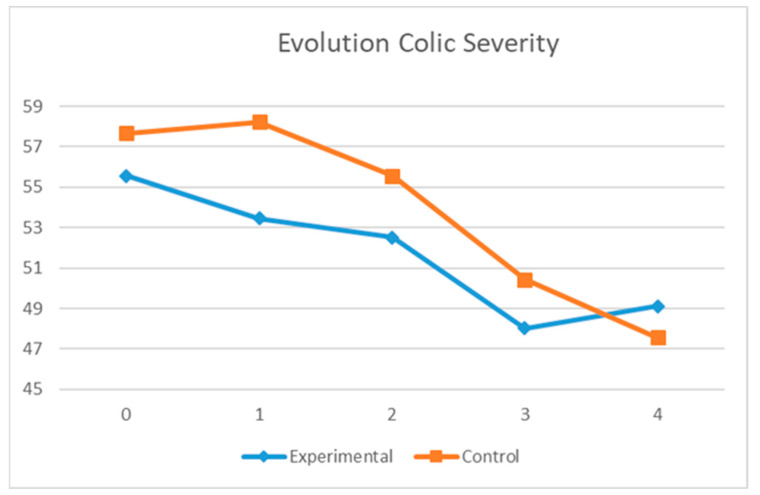
Repeated measures design of the evolution of colic severity.

Figure 2 shows that in the experimental group, the severity of colic had decreased at T1 (after treatment), but this was not the case in the control group. The two groups present clearly differing trends in this respect, especially in (T0–T1) and (T3–T4).

The repeated measures design revealed significant differences in the group–time relationship in (T3–T4) for the experimental group. However, there was no decrease in severity at T4, because the severity had already fallen to normal levels (<50) in T3. By contrast, the infants in the control group did not achieve these normal values until T4.

**Figure 3:**
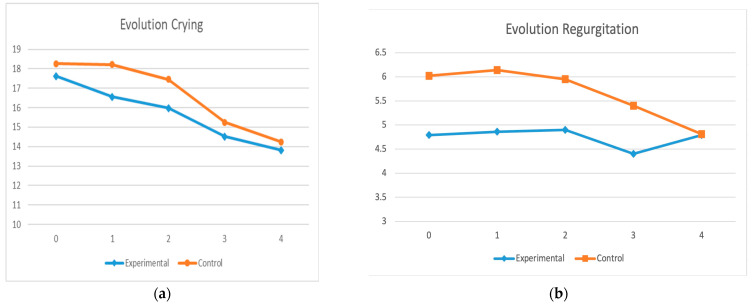

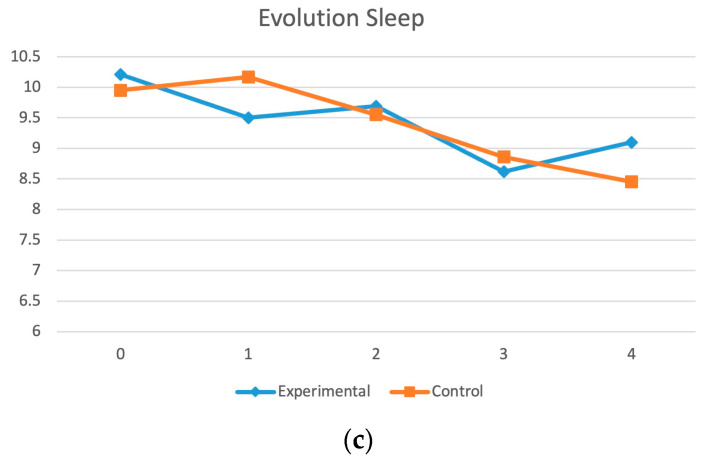
Evolution of the measurements of dimensions of questionnaire (**a**) evolution of crying (**b**) evolution of regurgitation (**c**) evolution of sleep.

As shown in Figure 3, the severity of various dimensions of colic varied significantly between the study groups, with more favourable values in the experimental group in every case, with these values becoming stabilised more rapidly, at the levels established as normal by the questionnaire. Within each group, there were no statistically significant differences in the variations observed at different times, although severity values decreased during the treatment.

## 4. Discussion

Our analysis of the study results shows that osteopathic manual therapy effectively alleviates the symptoms related to infant colic, particularly crying, regurgitation and interrupted sleep.

With respect to crying, the pathognomonic symptom of infant colic, the results were favourable after the first treatment and the improvement was maintained during the second week. In most cases, the parents reported a qualitative improvement in the baby’s condition after each intervention. These data are in line with Hayden [49], who reported a decrease in infant crying when cranial sacral therapy was applied, suggesting that cranial manipulation might reduce somatic afference to the nervous system. Browning [50] also observed benefits from osteopathic treatments in terms of reduced infant crying, although the differences between the intervention groups were not statistically significant. In the latter study, spinal manipulation was applied to one group and cranial therapy to the other. The benefits recorded in our own trial may have arisen from the application of osteopathic manual therapy targeting the structural, cranial and visceral spheres. In another related study, Miller et al. [51] obtained positive results, with less crying by the babies receiving treatment. These authors also analysed the impact of blinding the parents to the treatment provided. They found no differences between the groups, and therefore concluded that this blinding does not influence the effectiveness of osteopathic treatment. Nevertheless, we consider it important to avoid the possibility of bias in this regard; therefore, in our study, neither the paediatricians nor the families, although present, knew or were informed as to which group the children had been assigned. The effectiveness of the blinding was assured because the parents were unable to differentiate between the manual therapy procedures employed, as there was no spinal manipulation that could identify the treatment given to the intervention group. Moreover, the physiotherapist’s hands were in continuous contact with the baby, and continually changing position throughout the treatment, in both groups, whether or not the osteopathic manual therapy techniques were performed.

García-Marqués et al. [52] evaluated the effects of manual visceral therapy and massage on infants with colic. The treatment achieved positive results, with reduced crying, but the differences were not statistically significant. By contrast, in our study, with the same number of sessions, these differences were statistically significant throughout the follow-up probably because the treatment was provided in a more holistic way, i.e., it was not restricted to the visceral sphere.

However, our results differ somewhat from those obtained by Castejón-Castejón et al. [53], who also applied craniosacral therapy. These authors reported positive results in the reduction of crying and in the severity of infant colic, which were maintained throughout the 24 days of follow-up, during which treatment was carried out, ranging from one to three sessions depending on the severity of the condition. In our case, the differences in terms of crying or severity were not maintained after the second intervention (T3), although this may have been due to the fact that (according to the mothers), the improvement shown by the babies was greater in the days immediately following treatment, and the measurements were taken at one week after treatment. Therefore, the difference between our results and those of prior research may be explained by the separation required between sessions in order to maintain the benefits of osteopathic manual therapy or, alternatively, to divergences in the parents’ interpretation of the results achieved.

Driehuis et al. [43], in their systematic review and meta-analysis of manual spinal therapy in children, highlighted the difficulty of carrying out meta-analyses in this field because treatments cannot be compared. In consequence, they were unable to confirm that spinal mobilisation is an effective means of reducing the duration of crying due to infant colic. Our intervention, based on osteopathic manual therapy, includes cranial and visceral techniques that could be of greater benefit than the above-mentioned method by taking a more holistic approach.

On the other hand, our results are in line with those of Carnes et al., who conducted a meta-analysis of the use of manual therapy for infant colic [33] and reported a significant impact on reducing crying. Our finding that osteopathic manual therapy helps reduce crying time is also corroborated by Ellwood et al. [21], whose systematic review obtained moderate evidence that manual therapy reduced crying time and had no adverse effects. Nevertheless, these authors could not determine whether the improvement was due directly to the manual therapy or to other aspects of care.

With regard to the sleep variable, our results are qualitatively positive, in line with practically all the studies consulted, in terms of nocturnal sleep duration and continuity. In quantitative terms, although the differences observed were not statistically significant, the experimental group presented more favourable results at the post-treatment time points (T1 and T3). From this, we conclude that the therapeutic intervention described is optimal in this respect, obtaining benefits from the first session. This outcome was also reported by Castejón-Castejón [53]. Hayden [49] also reported significant improvements in babies’ sleep patterns after treatment. In our opinion, it is important to assess this variable because the discomfort produced by colic may influence sleep and rest.

Although the degree of regurgitation during infant colic is not usually measured in research studies, we believe this factor should be considered in the diagnosis and analysis of infant colic. According to our results, the initial quantitative improvement in this respect was only maintained until week 3. Nevertheless, this should be considered a positive outcome because at the final time point, the babies were three months old and by then the problem had resolved naturally in the control group. Moreover, the analysis by type of regurgitation revealed some improvement throughout the follow-up, in terms of decreased tension and enhanced functioning of the oesophageal sphincter following the visceral therapy applied to areas of abdominal tension. This finding suggests that osteopathic manual therapy may be a valuable non-invasive option for the treatment of symptoms related to regurgitation and gastro-oesophageal reflux.

The bibliography of studies related to osteopathic therapy for infant colic reveals considerable variability in the criteria and techniques applied, which should be taken into consideration in any analysis of the findings reported. Moreover, relatively few well-conducted randomised controlled trials have been conducted and this therapy is not systematically used for infant colic, despite the evidence of its benefits, benign nature and absence of side effects [33]. This assessment is corroborated by Cetinkaya [44], whose systematic review of complementary therapies for infant colic showed that although a variety of therapies have been proposed and many are effective in reducing crying without side effects, there is no consensus on their application as yet.

In order to resolve this heterogeneity, studies should seek to minimise the possibility of bias, thus underpinning the reliability of their findings. Furthermore, relevant focused information should be transmitted to paediatricians, midwives and others working in the field of infant health, to increase awareness of the benefits of using manual therapy to alleviate the symptoms of infant colic. In Spain, the disciplines of physiotherapy and osteopathy have evolved significantly, both academically and socially. The greater presence of osteopathy schools and postgraduate studies in this area has increased the number of specialised physiotherapists. Nevertheless, further attention to the issues we address is still required, both in clinical practice and in scientific research.

Our study is subject to certain limitations, as follows. Firstly, inter-observer validity criteria have yet to be established. This issue is quite widespread among studies of osteopathic manual therapy. Moreover, due to the variety of techniques used for the treatment of infant colic, it remains unclear which of them are most effective in resolving the symptoms. Our study was based on a protocol involving intervention in the visceral, cranial and structural spheres, taking a holistic osteopathic approach, in accordance with the multifactorial characteristics of infant colic. We could find a limitation in the study regarding the use of anti-colic products for infants.

A further problem is that we were unable to determine the adaptive physiological changes that are presumed to occur in the tissues when osteopathic manual therapy is applied, beyond the mechanical effects of manual contact.

In addition, our study did not incorporate home follow-ups to see how parents applied the information received during the intervention.

Finally, our analysis reveals certain lines of action that should be addressed in future research. One example would be to incorporate additional objective physiological measures to determine the level of inter-observer validity in the application of these measures or the blinding of the therapist.

## 5. Conclusions

Osteopathic manual therapy alleviates the crying, regurgitation and interrupted sleep that are typically related to infant colic. This treatment is effective from the first session and has no adverse side effects.

## Figures and Tables

**Table 1:** Baseline characteristics of babies in experimental and control group.

	Experimental (*n* = 42)	Control (*n* = 42)	*p* Value
**Weight**	3370 (770)	3372.5 (490)	0.38
**Weeks’ gestation**	39.45 (2.1)	39.75 (2.8)	0.20
**Sex**			
Male Female	22 (52.38) 20 (47.62)	29 (69.05) 13 (30.95)	0.11
**Order of siblings**			
Firstborn Second Third	22 (52.38) 17 (40.48) 3 (7.14)	18 (42.86) 18 (42.86) 6 (14.29)	0.49
**Type of feeding**			
Breastfeeding Formula Mixed	19 (45.24) 14 (33.33) 9 (21.43)	13 (30.95) 12 (28.57) 17 (40.48)	0.15
**Type of delivery**			
Vaginal (without complications) Scheduled caesarean section Instrumental vaginal (with complications) Emergency caesarean section	20 (47.62) 3 (7.14) 13 (30.95) 6 (14.29)	17 (40.48) 8 (19.05) 12 (28.57) 5 (11.90)	0.44
**Anti-colic products**			
Yes No	34 (80.95) 8 (19.05)	40 (95.24) 2 (4.76)	0.04

Quantitative variables: median (interquartile range). Qualitative variables: *n* (%).

**Table 2:** Between-group comparison of colic severity.

Colic Severity	Experimental	Control	*p* Value
Basal T0	55.5 (6.8)	57.6 (9.4)	0.43
T1	53.4 (10.3)	58.2 (9.5)	0.09
T2	52.5 (7.3)	55.5 (8.9)	0.13
T3	48.0 (12.5)	50.4 (10.9)	0.28
T4	49.1 (7.9)	47.5 (6.6)	0.55
*p* value *	G 0.18	T 0.00	GT 0.03

Md (Sd) T0 = baseline; T1 = 7 days after first intervention; T2 = 15 days after first intervention, 0 days after second intervention; T3 = 7 days after second intervention; T4 = follow-up at 3 months of age. *p* value = Mann–Whitney; *p* value * = repeat measures design; G = group; T = time; GT = group–time interaction.

**Table 3:** Between-group comparison and evolution of the questionnaire dimensions.

		T0	T1	T2	T3	T4	
**Sucking**	E C	3.6 (1.1) 3.9 (1.3)	3.6 (1.2) 3.6 (1.2)	3.5 (1.1) 3.4 (1.0)	3.1 (1.2) 3.1 (1.0)	3.2 (1.1) 2.8 (0.8)	G 0.77 T 0.00 GT 0.03
**Crying**	E C	17.6 (3.1) 18.2 (3.2)	16.5 (4.0) * 18.2 (3.4) * *p =* 0.03	15.9 (3.0) * 17.4 (3.4) * *p =* 0.04	14.5 (4.6) 15.2 (3.7)	13.8 (2.7) 14.2 (2.8)	G 0.10 T 0.00 GT 0.35
**Excretion**	E C	10.3 (2.9) 10.0 (3.1)	10.1 (3.0) 10.2 (3.2)	9.8 (2.6) 9.8 (2.8)	9.5 (3.2) 9.2 (3.1)	9.7 (2.5) 9.3 (2.5)	G0.74 T 0.00 GT0.78
**Eructation**	E C	4.3 (1.0) 4.9 (1.6)	4.3 (1.1) 4.9 (1.6)	4.2 (0.8) 4.8 (1.5)	3.9 (1.3) 4.3 (1.5)	4.3 (0.9) 4.1 (1.3)	G 0.12 T 0.00 GT0.01
**Regurgitation**	E C	4.7 (2.1) 6.0 (2.8)	4.8 (2.2) * 6.1 (3.0) * *p =* 0.05	4.9 (2.4) * 5.9 (2.6) * *p =* 0.05	4.4 (2.6) * 5.4 (2.6) * *p =* 0.04	4.7 (2.4) 4.8 (2.1)	G 0.06 T 0.00 GT 0.01
**Sleep**	E C	10.2 (2.8) 9.95 ± 3.636	9.5 (2.7) 10.17 ± 3.695	9.6 (2.2) 9.55 ± 3.387	8.6 (2.6) 8.86 ± 3.073	9.1 (5.4) 8.45 ± 2.411	G 0.95 T 0.00 GT 0.41
**Gas**	E C	4.6 (1.1) 4.6 (1.6)	4.3 (1.2) 4.7 (1.6)	4.2 (1.0) 4.5 (1.6)	4.8 (1.4) 4.1 (1.7)	4.0 (0.8) 3.7 (1.1)	G 0.56 T 0.00 GT 0.090

Md (sd), G = group T = time GT= group–time; * Significant differences in the Mann–Whitney test; E = experimental, C = control.

## Data Availability

Data for this study are available under considerable request.
